# The role of anchor objects in scene function understanding

**DOI:** 10.1038/s41598-025-04122-0

**Published:** 2025-06-23

**Authors:** Lea Alexandra Müller Karoza, Sandro Luca Wiesmann, Melissa Lê-Hoa Võ

**Affiliations:** 1 Neuro-Cognitive Psychology, Department of Psychology, Ludwig Maximilians University of Munich, Munich, Germany; 2 Department of Psychology, Scene Grammar Lab, Goethe University Frankfurt, Frankfurt, Germany

**Keywords:** Psychology, Human behaviour, Visual system

## Abstract

Throughout every day, we perform actions, and action information has been suggested to inform scene categorization. Here we hypothesise that actions also drive the hierarchical structure of many scenes, where anchor objects (e.g., stoves) predict the presence and position of local objects (e.g., pots) by dividing a scene in functionally distinct ‘phrases’. Specifically, we test whether the presence of anchor objects informs scene function understanding. In Experiment 1, participants matched an action word and a scene from which we either removed an action-related anchor object (REL), an action-unrelated anchor (UNREL) or a non-anchor object (RAND). Matching performance was impaired in REL compared to UNREL and RAND. Experiment 2 measured scene function activation more implicitly by priming a lexical decision task (LDT) on action words with the same stimuli (including an inconsistent condition: INCON, e.g., “cooking” in a bathroom). LDT performance was impaired after INCON and REL compared to RAND and UNREL primes. A control experiment showed that this effect was partly but not solely due to scene categorization. The results imply that understanding scene function is most closely tied to anchor objects directly relevant for actions whereas contextual scene information is not always sufficient to give rise to this understanding.

**Supplementary Information:**

The online version contains supplementary material available at 10.1038/s41598-025-04122-0.

As we go about our days, we carry out various actions to reach our goals. Many of these actions require us to manipulate the objects around us, e.g. squirting some toothpaste onto our toothbrush. A central aim of studying human scene understanding should therefore not only be to describe how humans perceive and categorize scenes and objects, but to characterize how we understand their functions and interact with our surroundings in a goal-directed manner^1^. Here, we specifically test the hypothesis that so-called anchor objects^2^ are relevant for understanding the action possibilities a scene offers to a viewer.

The objects contained in a scene are one possible source of action information. This notion of objects holding information about potential actions they afford was already established by Gibson^3^. He defined *affordances* as both the objects in an observer’s surroundings and the expectations raised about the range of actions possible based on an object’s functions. More recently, affordances have been defined as the relation between the features of our environments (e.g., objects) and our abilities^4^, while distinguishing physical and learned^5^, functional^6^ or even social affordances^7^. Notably, affordances are often studied in rather probabilistic terms, meaning that participants are asked about the likelihood of successful action completion^8^. Recently, research on affordances of single objects^3^ has been extended to higher-level functions of whole scenes^9^. Importantly, here we take a first step towards disentangling the contribution of object-level and scene-level information to the understanding of action possibilities in a scene. While this work builds on the classic concept of affordances, we will use the term “scene function” to describe goal-oriented actions that can be performed in a scene^9^. We hope this nomenclature avoids confusion and aligns with calls for more precise terminology and definition of the term *affordance* as an object-property^10^.

To integrate findings focusing on different levels at which actions are understood, a framework that allows us to separate the environmental context into smaller units while simultaneously understanding it as a holistic element is needed. Objects occur and co-occur in greater contexts which impacts our perception: borrowing concepts from language, these regularities can be referred to as *scene grammar*(^2,11,12^, for reviews see^13,14^). A scene, similar to a sentence, can be separated into phrases of objects (e.g., the shower, sink and toilet phrases in a bathroom). These phrases are assumed to have functional relevance and are organised in a specific way; we find so called *anchor objects* at the core of a phrase (i.e., the sink in the sink phrase). Around that anchor object, *local objects* cluster in specific ways (i.e., the soap on top of, the mirror above, and the small cabinet below the sink). These are usually smaller and easier to manipulate. This phrasal structure helps us during visual search, where anchors guide our attention to local objects^2,13^. Even when arranging rooms, anchors provide us with structure^11^. Additionally, this hierarchy follows the rules of scene semantics (i.e., which objects belong in a scene—one would not usually find a toaster in the living room) and scene syntax (i.e., where objects belong—one would not expect the toaster on the floor^15^).

Scene function understanding has been studied on different levels of this postulated hierarchy. One of the two approaches we focus on here regards scenes as a holistic environment, focussing on the highest level of the hierarchy (i.e., the scene), and suggesting that the actions we can perform in a scene are what dictate its category^9,16–18^. For example, a kitchen might generally be defined by the presence of a stove and fridge^9^, but these objects are also present in a store selling kitchen appliances. Nonetheless, we understand intuitively that a kitchen showroom is not the same as a kitchen because the actions afforded by a kitchen (i.e., “cooking”) are not afforded by the store, which might be defined by the action “shopping”^9^. From this follows that the scene’s category should also be informative of the actions one can carry out in such scenes. We will refer to this assumption as the *scene-level approach* to scene function understanding. A different view focusses on lower levels of the scene-hierarchy and assumes that assessing scene functions is tied to understanding the individual objects we manipulate to reach a goal^3^. We will refer to this as the *object-level approach*. Importantly, objects in scenes are organized in set relations^15^ and clustered according to functionality^11,19^. This relational information may provide an additional source of information for scene function understanding, which could be referred to as *phrasal-level approach*. In this project, we start by disentangling the scene and object levels of relevant information and investigate the influence of objects and scene context on scene function understanding, aiming to provide a nuanced exploration of their mutual dependencies.

Two related challenges arise from this clear separation. First, both approaches are limited as the scene-level approach does not explicitly consider object relations while the object-level approach disregards higher-level scene information. In isolation, the approaches cannot answer how scene context and object perception jointly impact and inform scene function understanding. While scenes hold information about object presence, they are more than a sum of the objects they contain^14,20,21^. Still, we typically interact directly with the objects present within the room; we can both segment a scene into its individual components as well as comprehend it as a cohesive whole. Therefore, considering the object-level approach in addition to the scene-level approach appears necessary to describe how scene functions are understood from visual input.

One of the challenges is that scene, object and function information are highly correlated^17^. Their processing appears to rely on correlated mechanisms; when estimating how similar scenes are with regards to various attributes, high correlations between scenes judged based on objects and scenes judged based on their functions are observed^22,23^. This suggests that the representation of scenes might include object as well as scene function information. Additionally, understanding the functions of objects enables us to systematically integrate them into our concept of scenes: when learning either features or functions of novel objects, participants located them more efficiently when they were tied to a function than when the description relied on features^24^. To tackle this challenge of interrelatedness, we assessed the role of object-level and scene-level information for scene function understanding by carefully manipulating the presence of different objects in the scenes. By comparing how removing action-related anchor objects, action-unrelated anchor objects and action-unrelated non-anchor objects affects scene function understanding, we can determine at which level functions are recognised. Similarly, while the original definition of affordances only considered the agent and the object acted upon^3^, more recent notions argue that understanding higher-level affordances—here defined as functions—also relies on contextual information, like other available objects, events happening around us, and the actor’s abilities^25,26^. For example, the original definition of affordances that only considered the agent and the object acted upon might, if extended to whole scenes, argue that the store selling kitchen supplies affords cooking because it contains stoves, which, by learned association, afford cooking. By considering contextual information (e.g., being in a ‘store’ rather than a ‘home’), the presence of available objects (e.g., multiple stoves but no utensils or ingredients), and ongoing events (e.g., conversations with salespeople), a viewer can correctly infer the function of the kitchen supply store. This further exemplifies how scene functions rely not only on object but also context and thereby scene information.

Conclusively, the scene-level approach suggests that scenes hold a defining, holistic functionality while the object-level approach implies a more direct link of functions to objects. As objects, however, generally occur in the context of scenes, we seek to explore how object presence in scenes as well as scene context impact scene function understanding. Specifically, existing research has not assessed the significance of a scene’s hierarchical structure in scene function comprehension. In three experiments, we investigated how different levels of a scene’s hierarchy impact scene function understanding.

As we assume that anchor objects divide a scene in functionally distinct phrases, we postulate that scene function understanding relies mainly on the perception of anchor objects and therefore hypothesize that scenes lacking anchors lead to impaired scene function understanding. In Experiment 1, participants performed an explicit matching task, indicating whether action words and scenes match. In Experiment 2, participants were primed with scene images and performed a lexical decision task (LDT) on action words afterwards. This enables the investigation of the role anchor information and scene context play in priming action concepts by measuring representational activations in the mental lexicon more implicitly. In both experiments, we used 3D rendered scenes from VR environments as stimuli from which we removed either anchor objects or random non-anchor objects (see Fig. 1). If scene function understanding primarily relies on the holistic scene context, removing different objects (anchors vs. non-anchors) from the scene should have comparable effects on scene function understanding. However, if functions are a more directly related to the affordances of individual objects, the identity of the removed object should differently impair scene function estimations based on the relatedness of the removed objects to the respective function. Additionally, if anchor objects are relevant for scene function understanding even beyond direct relatedness, removing action-unrelated anchor objects should impair scene function understanding more than removing non-anchors. Here, we hypothesize that scene function understanding is predominantly based on the directly related anchor object, with slower and less accurate matching of an action to a scene lacking the related anchor than to scenes lacking random objects. We further expect that scenes lacking anchors lead to impaired mental scene function activation, while this effect should be stronger for action-related than for action-unrelated anchors. Scenes lacking anchors are thereby expected to serve as less informative primes than scenes lacking random objects in an LDT.

**Fig. 1:**
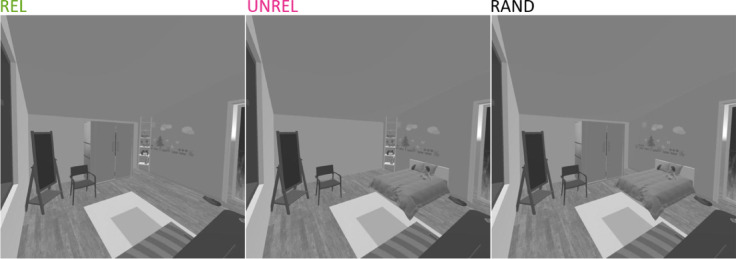
Example stimuli for the action “sleeping” depicting a bedroom scene in which either the bed (REL), wardrobe (UNREL) or a bookshelf (RAND) is removed.

## Experiment 1

In Experiment 1, participants were asked to report matches or mismatches between actions and scenes. We hypothesized that participants would be slowest and least accurate in their responses to scenes from which anchors related to the target action (REL) were removed (i.e., responding slowest to matching the action “sleeping” and the image of a bedroom lacking a bed). They would be faster and more accurate in their matching responses when anchors unrelated to the target action are removed from the scene (UNREL; i.e., “showering” and the image of a bathroom lacking a sink) and fastest and most accurate responding to scenes with random objects removed (RAND; such as mirrors, paintings, smaller shelves, or similar objects).

## Results

### Response time

**Fig. 2:**
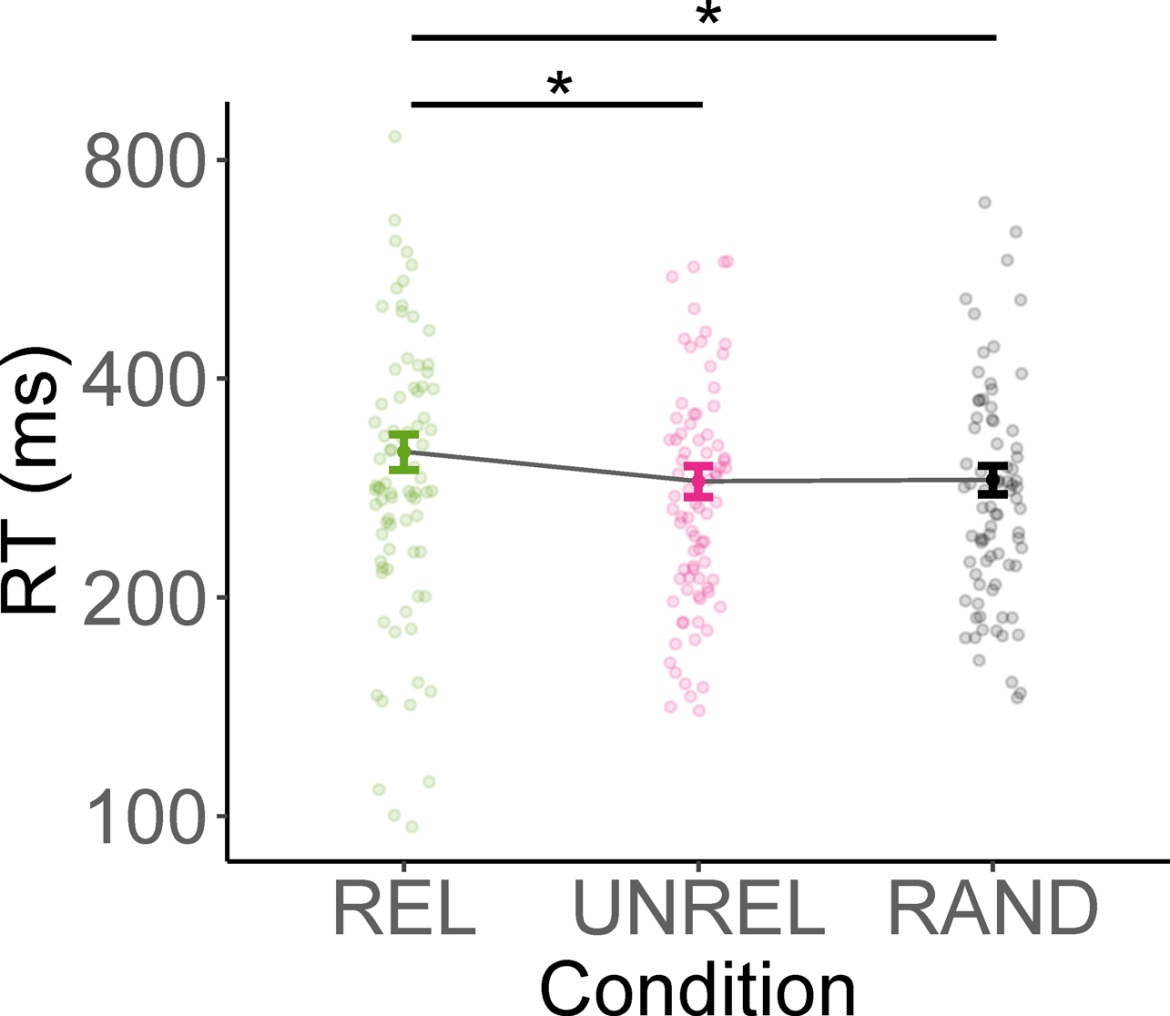
Average response times in the matching task of Experiment 1. *Note*. REL = action-related anchor object removed, UNREL = action-unrelated anchor object removed, RAND = action-unrelated non-anchor object removed. Error bars indicate confidence intervals computed using the correction proposed by Morey^27^. Dots indicate mean RT per participant. For increased readability, the y axis was log_2_-transformed.

In the final dataset, RTs ranged from 10 to 3603 ms. Mean RTs across object conditions are shown in Fig. 2, with fastest RTs for UNREL action-stimulus pairs (*M* = 285 ms, *SD* = 241), closely followed by RAND action-stimulus pairs (*M* = 288 ms, *SD* = 252), and slowest RTs for REL action-stimulus pairs (*M* = 312 ms, *SD* = 248). Results of the linear mixed-effects model predicting logRT are shown in Table S1 in the supplementary material, results of the Holm-Bonferroni corrected post hoc tests in Table 1.

**Table 1:** Results of Holm-Bonferroni corrected post Hoc estimations for the effect of object condition in the model predicting logRTs.

Contrast	β	*SE*	*p*
RAND vs REL	0.13	0.03	< .001
RAND vs UNREL	0.01	0.03	.851
REL vs UNREL	− 0.14	0.03	< .001

*p*-values are Holm-Bonferroni corrected for the three tests.

REL, action-related anchor object removed; UNREL, action-unrelated anchor object removed; RAND, action-unrelated non-anchor object removed.

As predicted, responses were significantly faster when the removed object was RAND or UNREL compared to REL. Notably, removing an anchor that was not related to the action did not impede matching responses. This shows that, when the action-related anchor is not visible, matching action words and scenes resulted in longer RTs compared to scenes in which the action related anchor was visible.

We also observed an effect of action typicality on logRT, (β = − 0.003, *p* < 0.001), showing that participants reported matches between scenes and action words faster when the actions were rated as more typical for the scene category they were matched with.

### Accuracies

**Fig. 3:**
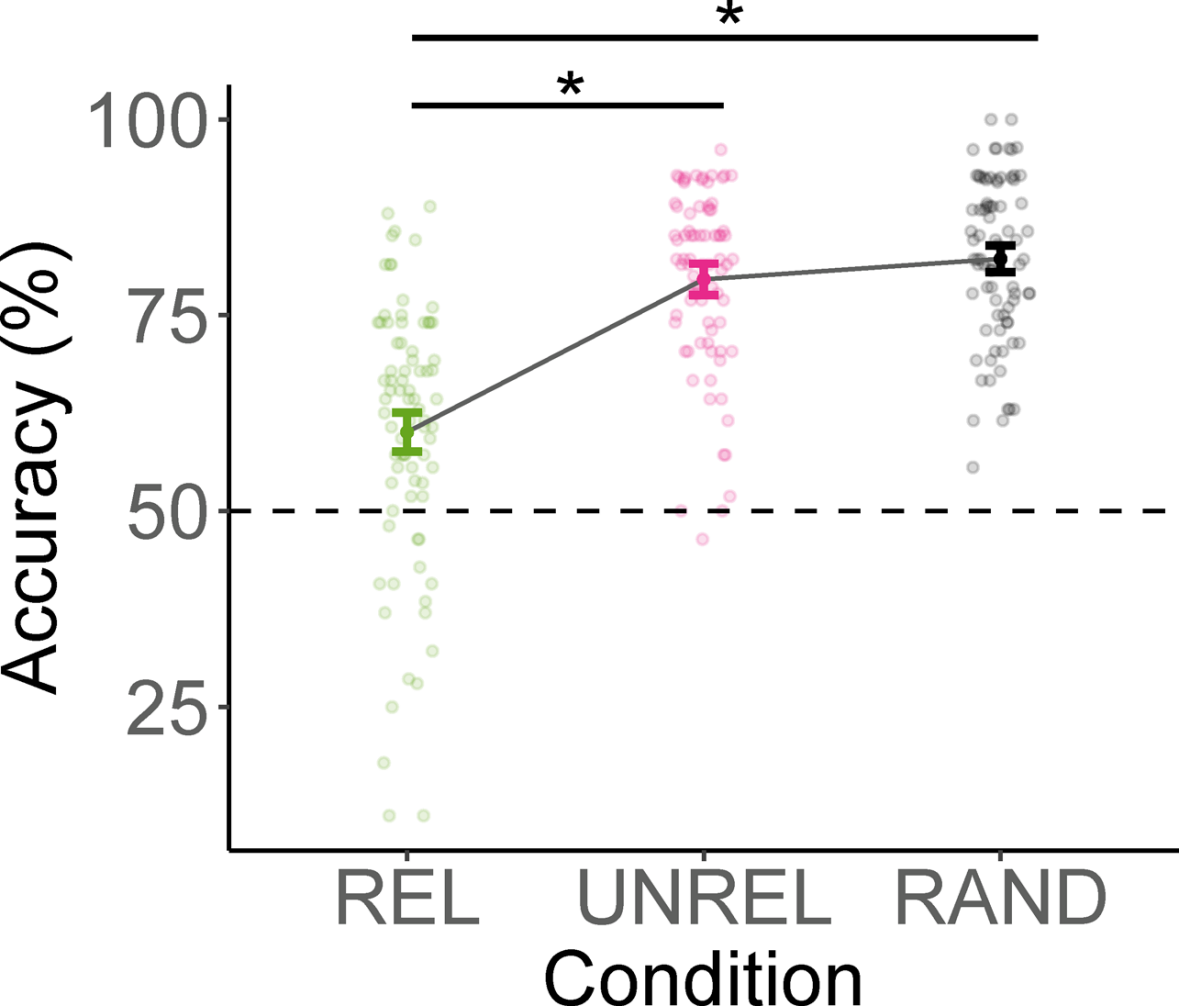
Average accuracy in the matching task of Experiment 1. *Note*. REL = action-related anchor object removed, UNREL = action-unrelated anchor object removed, RAND = action-unrelated non-anchor object removed. Error bars indicate confidence intervals computed using the correction proposed by Morey^27^.

Analogous to the findings in RTs, mean accuracies were lowest in the REL condition and highest in the RAND condition (Fig. 3). Results of the generalised linear mixed-effects model predicting accuracies are summarised in Table S2 in the supplementary material, details on post hoc computations are available in Table 2.

**Table 2:** Results of Holm–Bonferroni corrected post Hoc estimations for the effect of object condition in the model predicting accuracies in Experiment 1.

Contrast	β	SE	*p*
RAND vs REL	1.24	0.24	< .001
RAND vs UNREL	− 0.08	0.22	.720
REL vs UNREL	− 1.32	0.21	< .001

*P*-values are Holm–Bonferroni corrected for the three contrasts. Estimates are on the logit scale.

REL, action-related anchor object removed; UNREL, action-unrelated anchor object removed; RAND, action-unrelated non-anchor object removed.

Post hoc tests showed more accurate responses in the matching task for images from which random objects versus action related anchors were removed. The contrast between RAND and UNREL was not significant, indicating that accuracies did not differ between responses to stimuli missing unrelated anchors or random objects. Responses to images in the REL condition were significantly less accurate than to images in the UNREL condition. Increased action typicality significantly improved response accuracy; when actions were more typical for a scene category, responses in the matching task were more accurate. In line with our hypotheses on the impact of anchor presence on scene function estimations, this shows that when the action related anchor is visible (in RAND and UNREL), responses are more accurate compared to when it is removed from the scene (in REL), whereas we do not observe a difference between the removal of unrelated anchors or random objects.

### Typicality

Typicality ratings for the presented actions were generally high, with ratings above 90 (on a scale from 0–100) for all but three actions across the 16 actions used in the study. Mean typicality was lowest for “working” in bedrooms (*M* = 44.90, *SD* = 28.99), and “starting a fire” (*M* = 73.33, *SD* = 26.48) as well as “storing items” (*M* = 78.93, *SD* = 17.35) in living rooms.

## Discussion

This experiment investigated the effect of anchor object presence on scene function understanding. The results suggest that scene functions are strongly modulated by the objects visible in a scene. Removing an anchor relevant for the completion of an action impaired both the speed and accuracy of the matching response for that action. When other objects were removed, regardless of whether these were action-unrelated anchors or random non-anchor objects, responses were significantly more accurate and faster, and performance did not differ significantly between these conditions. These results are in line with our first hypothesis, that RT would be highest, and accuracy would be lowest when action related anchors are missing from a scene, suggesting that scene functions are at least in part conveyed at the level of action-specific anchors and not only via the scene context as a whole. This is congruent with Gibson’s classic notion of affordances as properties of individual objects^3^ and provides evidence for the object-level approach to scene function understanding.

As participants’ responses differed depending on the removed object, the effect of object removal in the scene is not universal but depends on the relation between actions and objects. Again, this does not go in hand with the notion of scene functions as a property of the scene as a whole^9^ but rather supports the idea of object-specific affordances^28^. Thus, we seem to rely on the objects either directly manipulated or in a broader sense connected to action completion more than on scene context in scene function understanding. This is particularly strengthened by the non-significant difference between removing unrelated anchors versus random objects—anchor absence does not appear to impact scene function understanding when the anchor is not used for the respective action.

In the second experiment, we sought to further investigate the importance of anchor objects for scene function understanding using a more implicit LDT paradigm. This is of interest as implicit scene affordance activation would suggest a fast and automatic process, possibly implying that processing of anchor presence automatically activates scene affordance understanding. If anchors impact scene function understanding, their presence or absence should differentially activate action words in the mental lexicon as seen in differences in RTs during an LDT^29^.

## Experiment 2

In the first experiment, we established that action-related anchor objects are important for fast and accurate assessment of scene function. To investigate whether this impact of anchor objects extends to a more implicit activation of scene function representations, we carried out a second study in which the same stimuli as used in Experiment 1 served as primes for an LDT on action words. Differences in responses across conditions during an LDT would suggest that the processing of anchor objects impacts scene function comprehension fast and automatically, such that activations of lexical entries in the mental lexicon are elevated by these primes to effect purely lexical decisions*.* We hypothesize that anchor absence will impair the mental representation and activation of scene functions, leading to poorer performance in the LDT when action words are primed by scenes without them. We expect most accurate performance following scenes containing the related anchor object and impaired performance when the related anchor was removed from the priming scene. We included semantically inconsistent scenes as uninformative control stimuli which should maximally impede LDT performance due to their semantic inconsistency^15^.

## Results

**Fig. 4:**
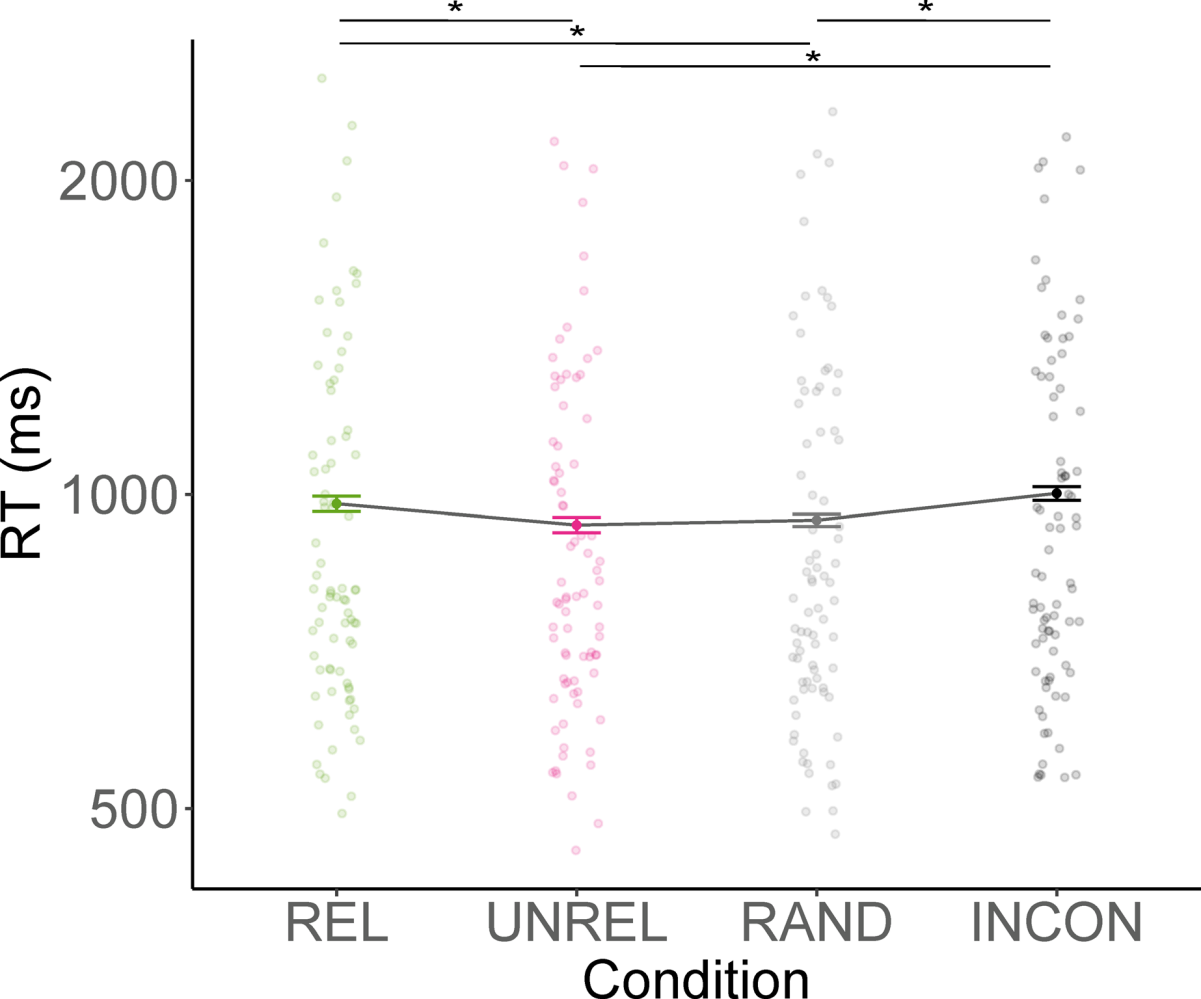
Mean response times across the four conditions in the LDT. *Note*. REL = action-related anchor object removed, UNREL = action-unrelated anchor object removed, RAND = action-unrelated non-anchor object removed, INCON = inconsistent scene category. Error bars indicate confidence intervals computed using the correction proposed by Morey^27^. For increased readability, the y axis was log_2_-transformed.

As accuracy in the LDT was at ceiling level, it was not analysed. RTs ranged from 16 to 6150 ms after removing a total of 3.11% of the trials due to either incorrect responses (1.27%) or response times more than three standard deviations from a participant’s mean (1.84%). Mean RTs across object conditions are shown in Fig. 4, with fastest RTs for UNREL action-stimulus pairs (*M* = 938 ms, *SD* = 553), followed by RAND (*M* = 946 ms, *SD* = 583), REL (*M* = 980 ms, *SD* = 590) and semantically INCON action-stimulus pairs (*M* = 1009 ms, *SD* = 572). Results of the linear mixed-effects model predicting logRTs are summarised in Table S3 and results of Holm-Bonferroni corrected post hoc tests in Table 3.

**Table 3:** Results of Holm–Bonferroni corrected post Hoc estimations for the effect of object condition in the model predicting logRTs in Experiment 2.

Contrast	β	*SE*	*p*
RAND vs REL	0.04	0.01	.006
RAND vs UNREL	0.00	0.01	.704
RAND vs INCON	0.06	0.02	.002
REL vs UNREL	− 0.04	0.01	.004
REL vs INCON	0.02	0.01	.219
UNREL vs INCON	0.06	0.02	.001

*P*-values are Holm–Bonferroni corrected for the six tests.

REL, action-related anchor object removed; UNREL, action-unrelated anchor object removed; RAND, action-unrelated non-anchor object removed; INCON, inconsistent scene category.

In line with the hypothesis that stimuli would be least effective in priming the LDT if action related anchors were removed from the scene, responses were slower in the REL condition compared to both the UNREL and RAND conditions. As in Experiment 1, RTs in the two conditions containing the action-related anchor (UNREL and RAND) did not differ significantly. Notably, there was no statistically significant difference in RTs between REL and INCON stimuli (the latter being assumed to be uninformative of the LDT), underlining the impact that removing action-related anchors has on the activation of scene function representations.

## Discussion

The results of Experiment 2 are in line with the hypothesis that scene functions are understood on the basis of the objects in a scene, thereby replicating the results of Experiment 1 in a task that involved less explicit function judgements. Participants were faster to respond in the LDT when it followed stimuli from a consistent scene that still depicted the related anchor than when the related anchor was either removed from the scene or when the entire scene was semantically inconsistent with the action. This suggests that action-related anchor presence substantially and specifically contributes to the activation or mental representation of scene functions. In contrast, scene functions are not as accessible when the anchor related to an action is removed from the scene, which is in line with the notion that object functions are specifically linked to the objects used for action completion^3,28^. Interestingly, while there was a descriptive difference in RTs between scenes lacking the action related anchor and scenes that are entirely semantically inconsistent with the action, they did not significantly differ. While this nonsignificant difference does not imply equality, it is interesting to note that a semantically consistent scene from which solely an action-related anchor was removed appears to have lost its ability to activate action concepts in the mental lexicon, thus not providing evidence in favour of scene-level function understanding.

The observed impact of removing action-related anchors on RTs suggests that high-level functions are more directly activated by the object used to carry out an action than by the scene as a whole. Objects appear to activate specific object-function representations, suggesting that we represent them in the proximate context we carry them out in (i.e., the *reachspace*^19^ or *phrase*^11^ rather than the whole scene). Additionally, performance on scenes lacking unrelated anchors did not differ significantly from performance on scenes lacking random objects. Thus, while the presence or absence of action-unrelated anchors might affect scene understanding, they do not seem to affect function perception on the scene-level, neither directly nor indirectly.

Generally, as the experimental procedure required participants to rate the fit between action words and the scene and therefore consciously processing the scenes before completing the LDT, we refrain from interpreting the mechanism at play as purely implicit. Yet asking participants to judge whether a letter string is a word or non-word implicitly measured the effects of anchors on the activation of action concepts via assumed activations in the mental lexicon. As a result, performance in the LDT described here likely reflects both implicit and explicit action processing.

Importantly, it is possible that impaired scene function understanding in Experiments 1 and 2 was simply an effect of impaired scene categorization due to removing diagnostic objects from the scenes. We therefore conducted a third study investigating the effect of anchor and random object removal on scene categorization performance. Recent studies suggest that both anchor objects^30^ and functions^9^ are diagnostic of scene category. If removing anchor objects also affects scene categorization, we cannot rule out the possibility that reduced scene function understanding in Experiments 1 and 2 is merely the result of impaired scene categorization. If, however, removing anchor objects from the scenes leaves scene categorization performance unaffected, this interpretation appears unlikely.

## Experiment 3

In the previous experiments, we showed a robust relation between anchor objects and the actions afforded by them. However, we cannot rule out that the effect of removing action-related anchor objects on scene function understanding is partly an artefact of impaired scene categorization leading to insufficient activation of the scene and thereby its functions. To investigate how the removal of anchor versus random object information impacted not only action but also scene understanding, we carried out this third experiment in which participants categorized the stimuli used in the previous experiments. Different categorization performance across conditions would suggest that stimuli are recognised as representatives of their category differently depending on which information is available. While the stimuli always depicted at least one anchor object (in REL, the action-related anchor was removed while the unrelated anchor was visible, the opposite was the case in UNREL), it is still possible that scene categorization performance is impaired when an anchor is removed (ANCH) compared to when random objects are removed (RAND). This would indicate that scenes lacking anchor objects are not understood as quickly and efficiently as scenes with intact anchor information. That could suggest that the effects found in previous experiments were not only a result of a close connection of anchor objects and the actions performed in the context of their phrase but also related to difficulties categorizing the scene itself.

## Results

### Reaction time

**Fig. 5:**
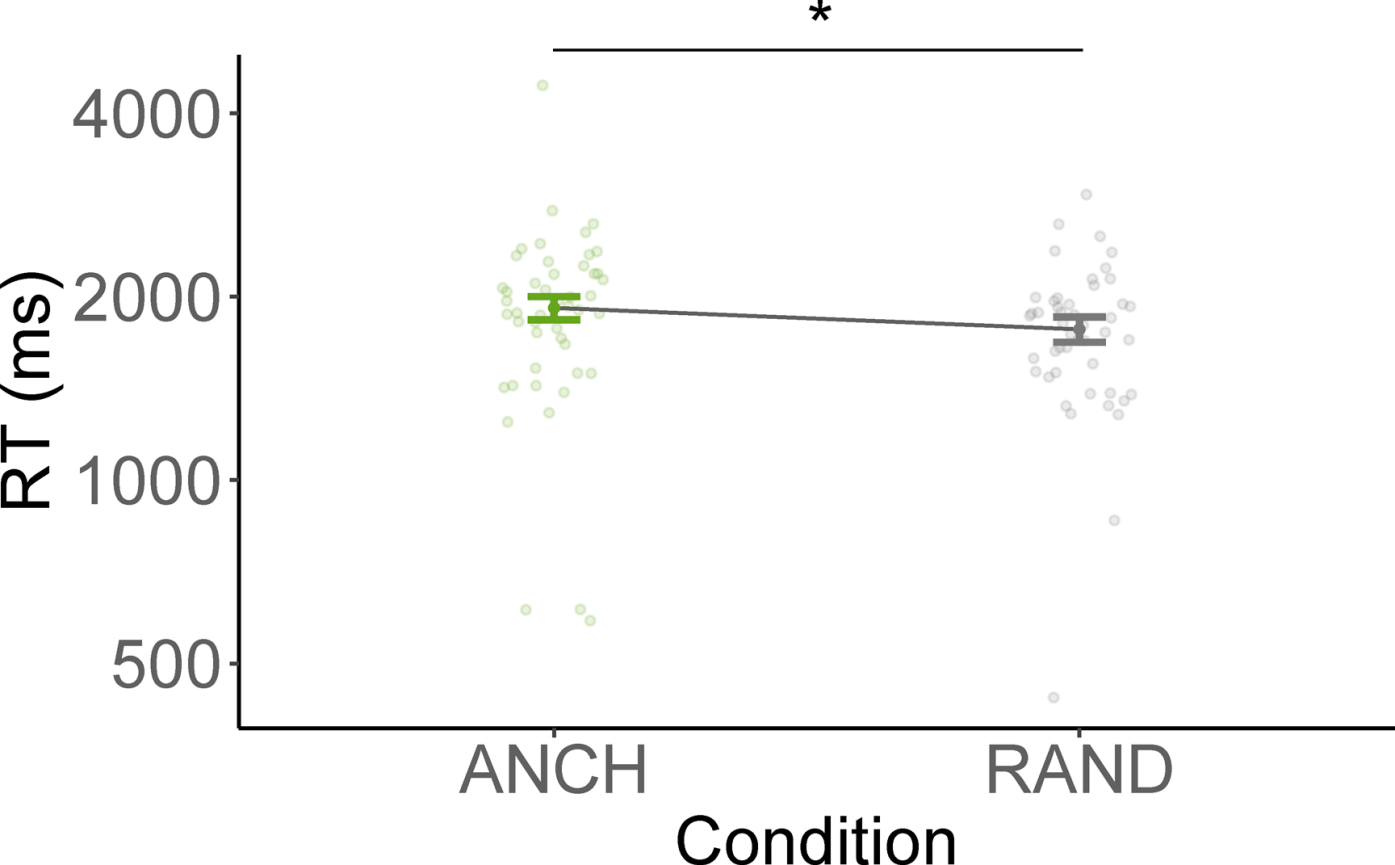
Mean response times across removed objects in scene categorization. *Note*. ANCH = anchor object removed, RAND = non-anchor object removed. Error bars indicate confidence intervals computed using the correction proposed by Morey^27^.

Correct RTs ranged from 109 to 8764 ms after removing a total of 47.36% of the trials due to either incorrect responses (46.50%) or response times that were either more than three standard deviations from a participant’s mean or slower than ten seconds (0.86%). The high rate of incorrect responses is consistent with previous findings from our group^30^ and probably due to the use of a brief presentation time (33 ms) in combination with a visual mask (100 ms, ISI 0 ms), and the use of relatively sparse, greyscale 3D rendered images with limited textural detail (see Figs. 1 and 7). Mean RTs across object conditions are shown in Fig. 5, with faster RTs for RAND (*M* = 1771 ms, *SD* = 860) compared to ANCH (*M* = 1907 ms, *SD* = 998) stimuli. Results of the linear mixed-effects model predicting logRTs are summarised in Table 4. In line with the hypothesis that stimuli would be categorized as exemplars of their category faster when all anchor information was available, responses were significantly faster when the removed object was RAND compared to ANCH.

**Table 4:** Results of the linear mixed effects model predicting logRT in Experiment 3.

Fixed effects	β	*SE*	*t*
Intercept	7.45	0.08	99.25
RAND vs ANCH	− 0.08	0.03	− 2.96

|*t*|> 2 is considered significant.

ANCH, anchor object removed; RAND, non-anchor object removed.

### Accuracies

**Fig. 6:**
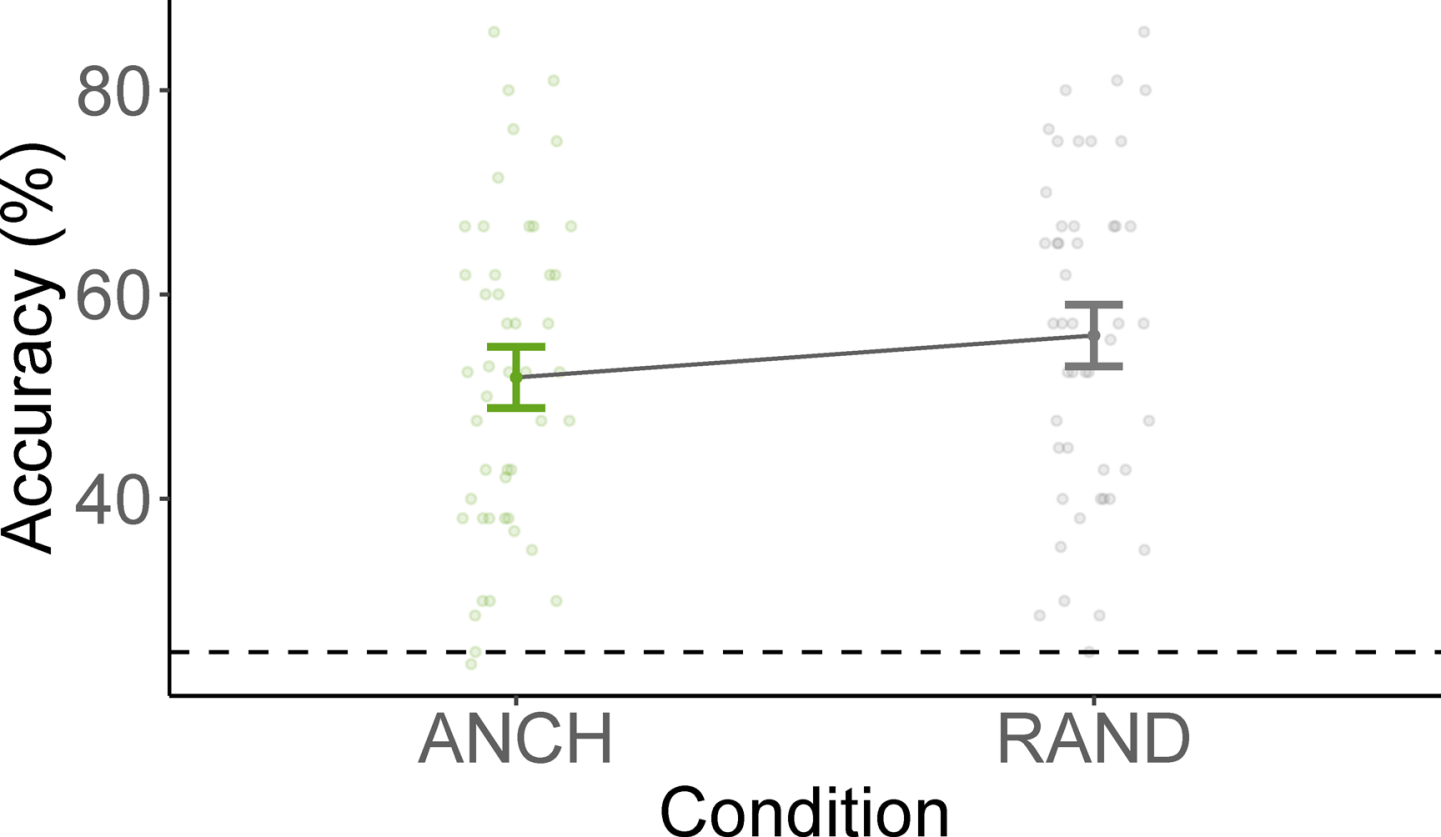
Mean accuracy across removed objects in scene categorization. *Note*. ANCH = anchor object removed, RAND = non-anchor object removed. Error bars indicate confidence intervals computed using the correction proposed by Morey^27^.

We excluded 2.33% of the data due to RTs being further than 3 standard deviations from participant mean or slower than 10 s. Results from the generalised linear mixed-effects model predicting accuracies are summarized in Table 5 and Fig. 6. Results suggest that removing anchor versus random objects does not differentially impact scene categorization performance in this experiment (but note that the *p* value was 0.054). A one-sided Wald z-test comparing the coefficients on the logit-scale to the logit of the chance level (25% in a 4AFC) suggests that both conditions elicit categorization performance significantly above chance-level (ANCH: *z* = 3.83, *p* < 0.001; RAND: *z* = 12.86, *p* < 0.001).

**Table 5:** Results of the generalized linear mixed-effects model predicting accuracy in Experiment 3.

Fixed effects	β	*SE*	*z*	*p*
Intercept	− 0.07	0.18	-0.37	.712
RAND vs ANCH	0.19	0.10	1.93	.054

ANCH, anchor object removed; RAND, non-anchor object removed.

### Supplementary analysis

To quantify the contribution of impaired scene identification to the effects of removing anchor objects on scene function understanding in Experiments 1 and 2, we computed mean accuracy scores for each image and then included these as an additional predictor in the models described for the respective previous experiments, comparing fits using the Likelihood Ratio Test. Importantly, while the initial experiments included stimuli in three conditions (“first anchor removed”, “second anchor removed,” and “random object removed”), the follow-up categorization study was limited to two conditions: one of the two anchor-removed conditions (either the “first” *or* the “second”) and the “random object removed” condition. This reduction was necessary for a balanced design including an equal amount of “anchor removed” and “random object removed” trials. However, it meant that a third of the data from the original stimulus set was excluded in the following analysis as we only have the categorization accuracy for half of the “anchor removed” stimuli used.

## Results

In Experiment 1, the inclusion of accuracy scores in the models predicting RTs and accuracies of matching performance improved model fit significantly but did not alter the overall pattern of effects, indicating that the claims made previously are robust even when accounting for scene categorization (see Tables S4 and S5 for model outputs and Tables S6 and S7 for post hoc test results). In Experiment 2, the inclusion of accuracy scores decreased the predicted difference in RTs between responses to actions primed with images missing the REL anchor versus images missing either the UNREL anchor or RAND objects and made these differences non-significant. Reaction times to actions primed with inconsistent scenes remained significantly slower than those primed with consistent scenes regardless of the removed objects (see Tables S8 and S9 for model outputs and post hoc test results).

## Discussion

Results of this experiment suggest that anchor objects can affect scene processing, as their removal significantly impaired the speed but not accuracy of scene categorization. Note, however, that the effect of the removed object on categorization accuracy was close to significance (*p* = 0.054), so we are careful in interpreting this as a null-effect and claiming an irrelevance of removed objects for the accuracy of scene categorization. Importantly, even when one anchor was removed from our stimuli, another one would always be visible. This might explain the non-significant effect of the removed object conditions in Experiment 3 (i.e., whether an anchor or a random object was removed). However, we observe that the removal of one anchor object—despite the presence of another—still significantly impaired the speed of scene categorization. We thereby conclude that scenes with removed anchor objects are categorized more slowly, and we cannot rule out that impaired scene categorization due to anchor absence impacted the results from Experiments 1 and 2.

When explicitly controlling for categorization by including the accuracy scores from Experiment 3 in the analyses for Experiments 1 and 2, we observed significantly improved model fits for both the matching task and the lexical decision task. Importantly, the inclusion of categorization in Experiment 1 did not affect the pattern or significance of results: although categorization was a significant predictor for matching performance, it did not explain the variance introduced by the different objects removed from the scenes. In Experiment 2, however, the inclusion of categorization accuracy did impact the pattern of results: while we previously found significant differences between conditions in which the related anchor was present (in UNREL and RAND) and those in which it was not (in REL and INCON), we only observed a significant effect of scene consistency (REL, UNREL and RAND versus INCON) when accounting for scene categorization. We assume this is because scenes depicting the related anchor allow us to infer the scene function directly via the object route, whereas scenes lacking the related anchor force us to use scene context to assess the associated scene functions. As we need more time to accurately infer scene category from stimuli without anchor objects (Experiment 3), the process of priming scene function information via the scene route might be delayed in Experiment 2. Thereby, we find partial support for the scene-level approach to scene function understanding. If scene functions were understood purely at object level, scene categorization should not have had an influence on results of Experiments 1 and 2. However, the LDT with the following consistency rating used in Experiment 2 may favour global scene processing, suggesting that the contribution of scene- versus object-level processing may depend on task demands. Additional support comes from the improved model fit when accounting for scene categorization in Experiments 1 and 2. While the strong link between objects and their associated actions primarily supports the object-level approach, the added explanatory power from scene categorization suggests that scene-level processing also plays a role in scene function understanding.

However, while impaired scene categorization explained the observed effects partially, it does not appear to be the sole explanation of scene function understanding. Both Experiments 1 and 2 showed significant differences between removing action-related and action-unrelated anchors (which were summarized in the ‘removed anchor condition’ in Experiment 3). If the reported effects were merely an artefact of scene categorization, it should be equal across all removed anchor conditions. Our results therefore suggest that anchor objects are relevant to understand the actions related to them beyond informing scene category. Taken together, our results thus provide evidence in favour of both object-level and scene-level function understanding.

## General discussion

In this study, we investigated the role different levels of a scene’s hierarchy play in scene function understanding by manipulating the presence of anchor objects in scene images. In Experiment 1, we were able to show that scene function understanding is based on anchor presence. When action-related anchor objects were removed from a scene, participants were less accurate and slower to match it to an action which could generally be carried out in scenes of the presented category. This shows that explicitly matching an action to a scene relies at least in part on the presence of anchor objects necessary for the completion of that action. Following this up with the more implicit LDT in Experiment 2, we observed that congruent scenes lacking the related anchor did not prime lexical access to action concepts as much as scenes that contained the action-related anchor. Taken together, we show that anchor objects involved in actions drive scene function understanding both when explicitly matching actions to scenes and also when participants performed a more implicit LDT on action words. As shown in Experiment 3, these effects can partially but not solely be explained by impaired scene categorization due to the removal of anchor information.

Functions have been postulated as defining properties of scenes and are among the best predictors for scene categorization^9,18^. In our experiments, we only find partial support for the reverse notion of scene-level function understanding. We investigated the impact of different levels of context information on scene function understanding and were able to show that it is strongest for scenes depicting anchor objects needed for an action. This suggests that we base our estimations of possible actions we can carry out in an environment on the anchors needed for that action rather than on the scene as a whole in which we typically carry out these actions. These results speak for an object-centric understanding of functions rather than holistic scene function representations. Importantly, we observed a mean accuracy of only around 60% in the matching task in Experiment 1 when action-related anchors were removed, which is comparably low—especially because most people should be able to match scenes and functions even if action-related objects are not directly visible (e.g., “showering” in the bathroom). However, we do not rule out the possibility that participants may have misinterpreted the prompt and based their response solely on the scene excerpt provided. The lack of a statistically significant difference between RTs for inconsistent scenes and scenes missing related anchors in the more implicit LDT in Experiment 2 points towards a similar notion. When the related anchor is removed from the scene, the whole scene seems to lose that particular function similar to a scene in which such action is hardly ever performed (i.e., a bathroom without a shower hardly activates the action “showering”, similarly to a kitchen scene). Importantly, as scene categorization explains parts of the discussed effects, we find partial support for the scene-level approach as well, even though this experiment overall suggests a more direct link of functions and objects.

In the object-level approach to scene function understanding, functions are considered to be closely related to the objects affording the respective actions^28^. Both Experiments 1 and 2 provide evidence for a close link between objects and their functions: Activation of scene function was driven by the presence of action-related anchors. This suggests that processing of objects used for action completion is a key determinant of scene function understanding. However, as initially elaborated, both functions^25,26,31^ and objects^15,32,33^ need to be understood within the context of the real world, which impacts how we perceive and represent them, informing recognition and memory. Specifically, we argued that objects, especially in man-made indoor scenes, are organised in hierarchies^2,11^. And while action-related anchor objects were important for scene function understanding, action-unrelated anchors were not, suggesting that action-relatedness is the crucial factor determining scene function understanding and not the anchor status itself. While anchor objects are assumed to be highly relevant for scene understanding due to their central role within the scene hierarchy, the scene’s reduced ability to prime scene function understanding without the action-related anchor speaks for function understanding at the object level. However, an open question remains whether action-related local objects are similarly suited to activate scene functions or whether action-related anchor objects are “special” beyond usually being large and easily detectable even in the periphery^13,14^.

As established above, the contribution of scene-level and object-level information to scene function understanding has, to our knowledge, not yet been systematically assessed. In our first two experiments, we showed that we primarily rely on action-related object information to understand a scene’s function, but when action-related object information is not available, we make use of the scene context to infer action possibilities in a scene. Notably, Experiment 3 suggests that this scene context information is, to an extent, also impaired by our manipulations of anchor presence in the scenes. Including the scene categorization accuracy from Experiment 3 in the models of Experiments 1 and 2 revealed distinct effects but was unable to account for all observed differences across conditions, aligning with the idea that scene function understanding relies on both objects and scene context (e.g., “showering” can be inferred by the presence of a shower and from the context of a bathroom). Removing the action-related anchor may slow RTs because it forces reliance on contextual cues, like scene-level-information, to infer scene functions, and Experiment 3 showed that scene categorization itself is slower when an anchor is removed. In contrast, removing action-unrelated anchors or non-anchors leaves the more direct object-route intact, allowing faster responses. Function understanding therefore appears to be flexible and can adapt to the available information, showing that conscious and deliberate function judgments, as tested in Experiment 1, are seemingly robust to categorization difficulties, while more implicit tasks such as lexical decision rely more on intact scene categorization when action-related object information is missing.

Importantly, the experiments reported here only allow us to compare the relative utility of action-related und unrelated anchors as the absence of one anchor object was always paired with the presence of a different anchor. To investigate the role of action-unrelated anchor objects in driving scene function understanding in more detail, future studies could compare stimuli containing action-unrelated anchors to a baseline condition in which both related and unrelated anchors are absent. This would allow testing the incremental utility of action-unrelated anchor objects in comparison to the non-anchor scene context alone and thus inform us which contextual information (action-unrelated anchor objects, global scene context) informs scene function understanding in the absence of action-related anchor objects. An additional approach could be the investigation of the impact object-level and scene-level information have on function understanding in more naturalistic scenes. While the 3D rendered scenes we used here provide us with full experimental control, they are distinctly different from real photographs, containing fewer objects, textures and possibly being processed differently. Moreover, classic affordances are often measured in more probabilistic terms^8^. Our approach of using matching-performance and an LDT to measure scene function is unconventional and might thereby be harder to relate to existing literature. However, we believe that the mixture of both an explicit and a more implicit task allows us to make more valid claims about the representation of scene functions. As implicit understanding tends to be automatic and fast, but we also act in planned manners, testing scene function understanding implicitly and explicitly appears to cover two ecologically relevant characteristics of functions.

In sum, the understanding of scene functions relies on the perception of anchor objects used for action completion, indicating that we primarily rely on the object-level route to scene function understanding. While scene context contributes to some degree, our findings indicate that objects predominantly drive scene function understanding. Conclusively, the direct link of scene functions and the anchors we use when carrying out actions opens the door for a more fine-grained investigation of scene functions and how we represent the action possibilities in our surroundings.

## Methods

### Experiment 1

#### Participants

Participants were recruited via Prolific (www.prolific.com) [04.07.2024]. Eighty native German speakers (age *M* = 29; range = 19–40, 61 women, 19 men) were included in the final analysis. A power analysis conducted on pilot data by 5 participants using the package *mixedpower* v0.1.0 for R^34^ suggested a power of > 0.80 for detecting a significant contrast of random objects versus related anchors being removed at this sample size. Before data analysis, one participant was excluded due to failing to identify at least more than half the mismatches between actions and scenes. The participants received 2.70 GBP as compensation for the 18-min experiment via Prolific. All participants confirmed having normal or corrected-to-normal vision and gave informed consent. We followed Goethe University’s guidelines for experimental studies with human participants. The experimental protocol was approved by the Goethe University’s Human Research Ethics Committee (Approval ID: 2014-106R1). We confirm that all methods used across the three experiments were performed in accordance with the relevant guidelines in the Declaration of Helsinki.

#### Stimuli

The forty-two stimuli depicted indoor scene excerpts from the following categories: eight bathrooms, eight bedrooms, sixteen kitchens and ten living rooms. Stimuli were taken from a previous study^35^ and adapted. They depicted two anchor objects selected by the first author based on the definition as objects that predict the spatial locations of other objects within a scene^2^ and anchorness ratings^30^ to ensure similar effects across categories and stimuli. The stimuli were 3D rendered scenes, manipulated by removing either anchor or random objects. Additionally, stimuli were resized to 512 × 512 pixels, and mean luminance and contrast were equalized across all stimuli using the SHINE toolbox^36^.

Action words were selected based on the anchors present in the unmanipulated scenes, with each word being directly related to one of the anchor objects (e.g., if a bed and a wardrobe were removed from a bedroom stimulus, the respective actions would be “sleeping” and “getting dressed”). The word selection process was guided by intuitive associations and later validated using the typicality ratings of category-action pairs. The experiment was carried out in German.

#### Procedure

The experiment was carried out online using jsPsych^37^, in which participants were assigned to one of nine counterbalanced stimulus lists allocating the object conditions to actions. A stimulus existed in three object conditions (1, anchor one removed, 2, anchor two removed, or 3, random objects removed) and could be paired with three verbs (1, action related to anchor one, 2, action related to anchor two, or 3, semantically unrelated distractor action). The pairing of stimuli and actions as well as their location in the blocks was determined by the nine counterbalancing lists (see Figure S1 in the supplementary material). Each participant saw every stimulus in one of the three object conditions three times, paired with a different action word but in the identical object condition. This resulted in a total of 126 trials over three blocks.

**Fig. 7:**
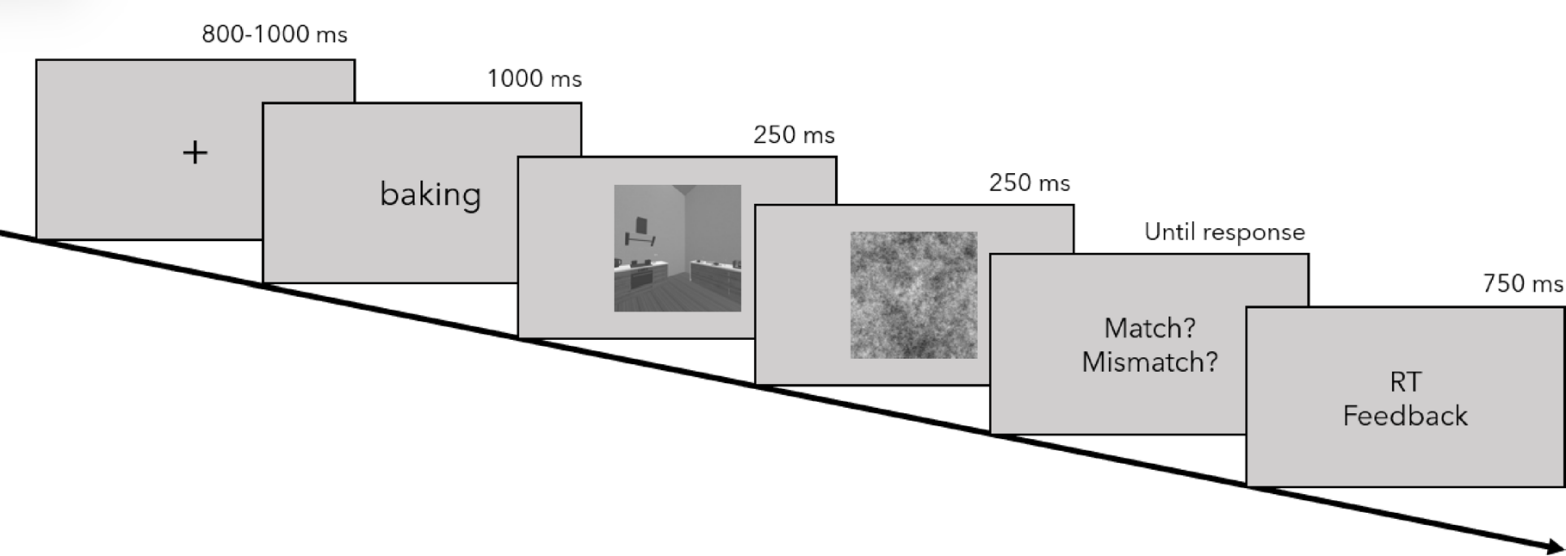
Trial sequence for the three experimental blocks.

In block one, stimuli were presented with one of the three possible action words they could be paired with. After all 42 stimuli were shown once, they were presented in the same object condition but paired with the remaining verbs. During the trial sequence initiated with a fixation cross, an action verb was presented for 1000 ms, followed by a stimulus and a pink-noise mask for 250 ms each. After that, participants decided whether the action word and the stimulus matched and received feedback on their reaction time to motivate fast responses (see Fig. 7 for trial sequence). After the three experimental blocks of the matching task, participants reported how typical the actions were for each of the scene categories they were paired with. Participants completed this task for all scene category-action pairings, including the inconsistent ones. Responses were given on a slider from 0–100. Responses were not timed during this task.

### Statistical analysis

Data were cleaned by removing RTs more than three standard deviations slower than the participant’s mean or faster than 10 ms from the subset of accurate trials to exclude implausibly quick responses and ensure applicability of the log-function. This resulted in removal of 28.79% of trials for the model predicting logRTs because participants responded too slowly (1.59%), too fast (2.02%), or inaccurately (25.18%) and removal of 3.61% of trials in the model predicting accuracy, as inaccurate trials were not removed from that subset of data. The 28.79% removed trials in the sub-data frame for calculations of RT is a substantial amount of data, which can, however, be explained by the fact that participants oftentimes identified scenes as mismatches of actions when the related anchor was absent (i.e., when the sink was removed and the target action was *brushing teeth*). We log-transformed RTs to ensure normally distributed data. The linear mixed-effects model predicting logRTs and the generalized mixed-effects model predicting accuracies are specified in Eq. (1) and Eq. (2).

1 $$\text{logRT}=\text{Condition }+\text{ Typicality }+ (1 |\text{ Participant})$$

2 $$\text{Accuracy}=\text{Condition }+\text{ Typicality }+ (1 +\text{ Condition }|\text{ Participant})+ (1 +\text{ Condition }|\text{ Image})$$

Condition refers to the semantic relatedness of action word and the removed object (REL, UNREL, RAND) with RAND as the reference category. Typicality was included to account for the interindividual differences in the participant’s perceived fit between the action and scenes of the category they were presented with. The analyses were conducted using RStudio v4.3.0^38^. We used the package tidyverse v2.2.0^39^ for data cleaning, lme4 v1.1-33 for the mixed-effects models^40^, emmeans v1.8.7 for post hoc computations^41^ and ggplot2 v3.4.2^42^ for data visualization. The random effects structure was initially specified to be maximal^43^ and was subsequently simplified by removing terms that did not significantly decrease the goodness of fit^44^.

## Experiment 2

### Participants

Participants were recruited via Sona Systems at Goethe University or through word of mouth. Eighty participants (age *M* = 24; range = 19–39, 62 women, 18 men) were included in the final analysis. In a power analysis on pilot data by 5 participants, we confirmed that mirroring this sample size from Experiment 1 had a power of > 0.80 for both the INCON vs. RAND as well as the REL vs. RAND contrast. Before data analysis, eight participants were excluded as they showed lower accuracy than two SDs from mean accuracy. Students of psychology at Goethe University received course credit as compensation for the 20-min experiment, no other form of compensation existed. All participants confirmed having normal or corrected-to-normal vison and gave informed consent. As before, we followed Goethe University’s guidelines for experimental studies with human participants and the experimental protocol was approved by the Goethe University’s Human Research Ethics Committee (Approval ID: 2014-106R1).

### Stimuli

The stimuli were identical to the ones used in the first study. For this experiment, we generated pseudowords using Wuggy^45^. Pseudowords were created to match properties of the action words such as syllable count and word length while taking the target language (German) into account. We included the list of all actions used as experimental trials in the study and chose 42 unique strings of letters from the list of pseudowords. These were chosen based on their perceived closeness to words in the German language, including their pronounceability and how obviously they could be identified immediately. Stimulus-action pairings existed on four levels of semantic relatedness: the action word was preceded by a scene from which we removed a related (REL) or unrelated (UNREL) anchor object, a random object (RAND), or by a semantically inconsistent scene (INCON).

### Procedure

The experiment was carried out online using jsPsych^37^, in which participants were assigned to one of twelve counterbalanced stimulus lists allocating the object conditions to verbs. A stimulus existed in three object conditions (1, anchor one removed, 2, anchor two removed, or 3, random objects removed) and could be paired with four strings of letters, while three of those consisted of real verbs of the German language (1, action related to anchor one, 2, action related to anchor two, or 3, semantically unrelated action) and the fourth was a pseudoword. The pairing of stimuli and actions as well as their location in the blocks was determined by the twelve counterbalancing lists following the same rationale as in the first experiment (see Figure S1 in the supplementary material). Each participant saw every stimulus in one of the three object conditions four times, paired with a different action word or string of letters, resulting in a total of 168 trials over four blocks. Out of those, 25% of trials included pseudo-words, another 25% of trials included inconsistent scene-action pairings, and the remaining 50% of trials included both consistent actions for the scene.

**Fig. 8:**
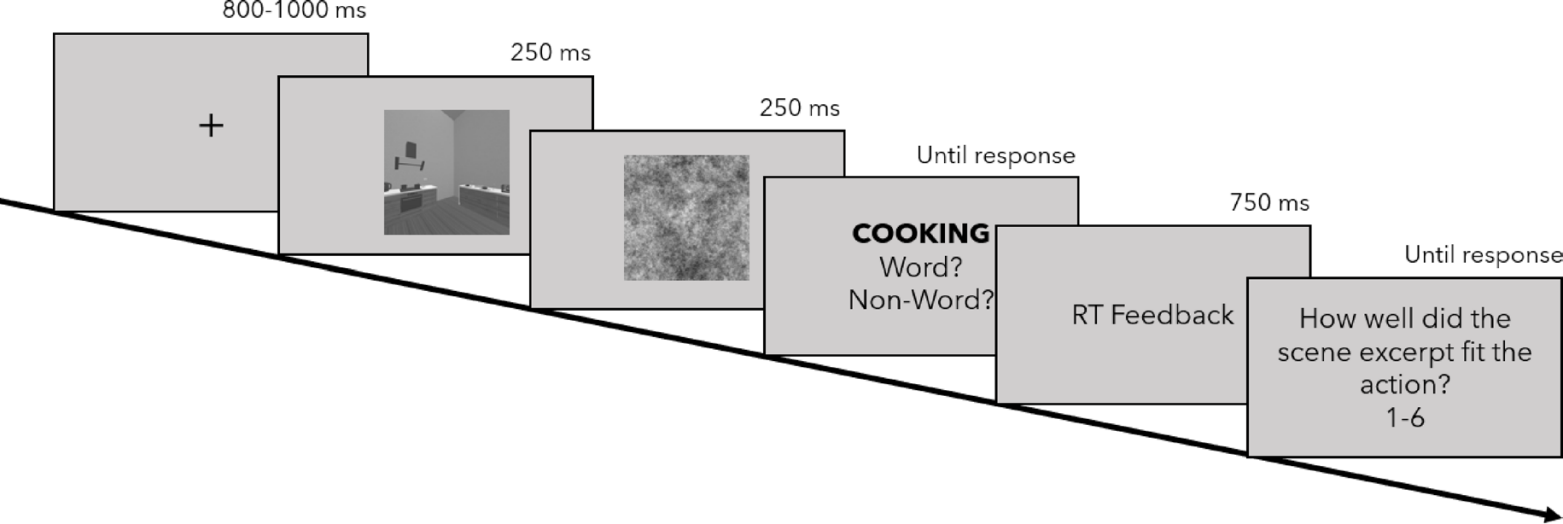
Trial sequence for the four experimental blocks in Experiment 2.

Each trial started with a fixation cross, followed by the presentation of a stimulus and mask for 250 ms each. Subsequently, the LDT was performed on actions or pseudowords in a two-alternative forced choice task (2AFC). Participants received feedback on their RTs but not the accuracy of their judgement. After each trial, participants indicated the fit between the stimulus they were primed with and the action or pseudoword on a scale from 1–6 to ensure that the priming stimulus would not be ignored (see Fig. 8). Following the four experimental blocks including the LDT for all strings of letters a stimulus was presented with, participants answered how typical they thought an action was for the category it was presented with on a slider response between 0–100. This was repeated for all verbs regardless of semantic relatedness but not for the pseudowords.

### Statistical analysis

Data were cleaned by removing trials with incorrect responses and with RTs more than two standard deviations slower than the mean or faster than 50 ms. Further, participants were excluded based on their inability to identify pseudowords with an accuracy of less than two standard deviations from mean accuracy. We transformed RTs into logarithmic values to achieve normal distribution for the statistical analysis. The linear mixed-effects model’s architecture is specified in Eq. (3). As accuracy in the LDT was at ceiling level, it was not analysed.

3 $$\text{logRT}=\text{Condition }+\text{ Length }+ (1 +\text{ Condition}+\text{Length }|\text{ Participant}) + (1 +\text{ Condition}+\text{Length }|\text{ Image})$$

Fixed effects for the condition of semantical relatedness (REL, UNREL, RAND, INCON) and word length are included. Word length is included as a fixed effect to account for the increased task difficulty in case of longer words. We decided against the inclusion of action-typicality as a predictor in the model as that hindered convergence and word length posed more relevant factor in the context of a lexical decision. Additionally, we include random slopes for the predictors and random intercepts for each participant and image. Software for data analysis was identical to Experiment 1.

## Experiment 3

### Participants

Participants were recruited via Sona Systems at Goethe University or through word of mouth. Forty-seven participants (age *M* = 22.5; range = 18–34, 32 women, 15 men) were included in the final analysis. Before data analysis, three participants were excluded as they showed lower accuracy than two SDs from mean accuracy. Students of psychology at Goethe University received course credit as compensation for the 10-min experiment, no other form of compensation existed. All participants confirmed having normal or corrected-to-normal vison and gave informed consent. Again, we followed Goethe University’s guidelines for experimental studies with human participants and the experimental protocol was approved by the Goethe University’s Human Research Ethics Committee (Approval ID: 2014-106R1). 

### Stimuli

The stimuli were identical to the ones used in the previous experiments. Due to the different task (see Procedure), the conditions REL and UNREL were collapsed into the ANCH condition, indicating that an anchor was removed from the scene. The RAND condition remains identical.

### Procedure

**Fig. 9:**
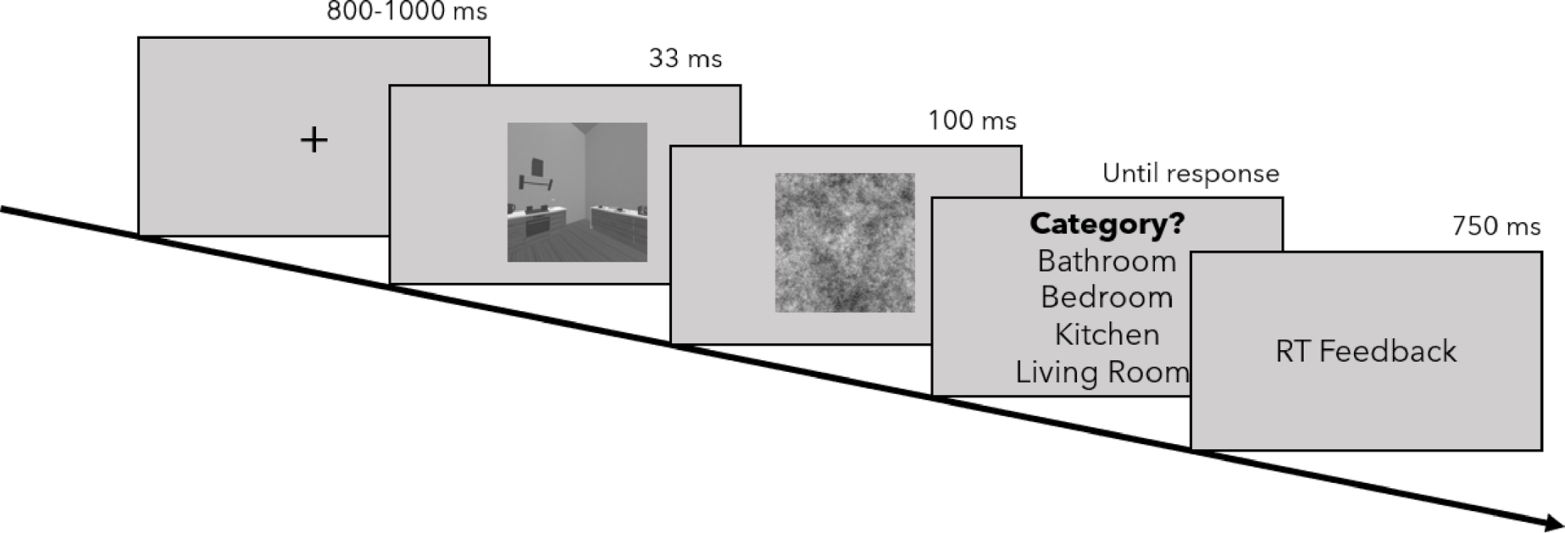
Trial sequence for the two experimental blocks in Experiment 3.

Like the previous studies, this experiment was carried out online using jsPsych^37^, in which participants were assigned to one of two counterbalanced stimulus lists, deciding which stimuli a participant saw in the ANCH versus the RAND condition. Each trial started with a fixation cross, followed by the presentation of a stimulus for 33 ms and mask for 100 ms. Subsequently, the participants categorized scenes as either bathrooms, bedrooms, kitchens or living rooms in a four-alternative forced-choice (4AFC). They received feedback on their RTs but not the accuracy of their judgement (see Fig. 9).

### Statistical analysis

Data were cleaned by removing trials with incorrect responses and with RTs more than three standard deviations slower than participant mean or faster than 10 ms. For the analysis of accuracy, data was only cleaned based on RTs and not correct responses. We transformed RTs into logarithmic values to achieve normal distribution for the statistical analysis. The linear mixed-effects model predicting logRTs and the generalized mixed-effects model predicting accuracies are both specified in Eq. (4).

4 $$\text{y}=\text{Condition }+ (1 |\text{ Participant}) + (1 |\text{ Category}/\text{Image})$$

Note. The model predicting logRT allowed a nested structure of images within scene category while the glmer predicting accuracy showed a singular fit with that variable. As the model including random slopes of the effect of the removed object did not fit the data significantly better than the model excluding it, we restricted the random effects structure for more robust results.

## Electronic supplementary material

Below is the link to the electronic supplementary material.

Supplementary Material 1

## Data Availability

The datasets generated and analysed during the current study are available on osf.io/wqan6.
