# Supplementary material for: The role of anchor objects in scene function understanding
Source: Sci Rep. 2025 Jun 23;15:20247. doi: 10.1038/s41598-025-04122-0 (PMC12185685; doi:10.1038/s41598-025-04122-0)
Supplement: Supplementary file 1 — Supplementary Material 1 [file 41598_2025_4122_MOESM1_ESM.pdf]

The Role of Anchor Object Presence in Scene Function Perception

Supplementary Material

Lea Alexandra Müller Karoza[1,2]*, Sandro Luca Wiesmann[2], & Melissa Lê-Hoa Võ[1,2]

[1]Neuro-Cognitive Psychology, Department of Psychology, LMU Munich, Germany

[2]Goethe University Frankfurt, Department of Psychology, Scene Grammar Lab, Germany

Contact via: MuellerKaroza@psych.uni-frankfurt.de

Table S1

Results of the Linear Mixed Effects Model Predicting logRT in Experiment 1

Fixed Effects	β	*SE*	*t*
Intercept	5.60	0.10	54.58
RAND vs REL	0.13	0.03	4.08
RAND vs UNREL	-0.01	0.03	-0.19
Typicality	0.00	0.00	-3.25

Note. Random intercepts for participants and images are allowed and random slopes for both predictors. $|t| > 2$ is considered significant. RAND refers to images with removed random objects, REL indicates images with action related anchors removed, and UNREL indicates images with action unrelated anchors removed.

Table S2

Results of the Generalized Linear Mixed-Effects Model Predicting Accuracy in Experiment 1

Fixed Effects	β	*SE*	*z*	*p*
Intercept	0.09	0.26	0.33	.744
RAND vs REL	-1.24	0.24	-5.09	< .001
RAND vs UNREL	0.08	0.22	0.36	.720
Typicality in percent	1.87	0.23	8.17	< .001

Note. Accuracies are transformed to logits. To ensure convergence of the model, typicality was transformed from its original scale of 0-100 to percentages between 0-1. RAND refers to images with removed random objects, whereas REL and UNREL refer to images with removed anchors that are related or unrelated to the action, respectively.

Table S3

Results of the Linear Mixed Effects Model Predicting logRT in the LDT in Experiment 2

Fixed Effects	β	*SE*	*t*
Intercept	6.62	0.04	147.32
RAND vs REL	0.03	0.01	3.10
RAND vs UNREL	0.00	0.01	-0.38
RAND vs INCON	0.06	0.02	3.56
Word Length	0.01	0.00	5.71

Note. Random intercepts for participants and images are allowed and random slopes for both predictors. $|t| > 2$ is considered significant. RAND refers to images with removed random objects, REL indicates images with action related anchors removed, UNREL indicates images with action unrelated anchors removed, and INCON refers to semantically inconsistent action-stimulus pairs.

Table S4

Results of the Linear Mixed Effects Model Predicting logRT in Experiment 1 Including the Accuracy Scores From Experiment 3

Fixed Effects	β	*SE*	*t*
Intercept	5.69	0.12	45.68
RAND vs REL	0.13	0.04	3.18
RAND vs UNREL	-0.01	0.04	-0.33
Typicality	0.00	0.00	-2.57
Accuracy	-0.17	0.08	-2.19

Note. Random intercepts for participants and images are allowed and random slopes for both predictors. $|t| > 2$ is considered significant. RAND refers to images with removed random objects, REL indicates images with action related anchors removed, and UNREL indicates images with action unrelated anchors removed.

Table S5

Results of the Generalized Linear Mixed-Effects Model Predicting Accuracy in Experiment 1 Including the Accuracy Scores From Experiment 3

Fixed Effects	β	*SE*	*p*
Intercept	-1.93	0.35	<.001
RAND vs REL	-1.18	0.26	< .001
RAND vs UNREL	0.27	0.26	.304
Typicality in percent	2.16	0.26	< .001
Accuracy	3.15	0.43	<.001

Note. Accuracies are transformed to logits. To ensure convergence of the model, typicality was transformed from its original scale of 0-100 to percentages between 0-1. RAND refers to images with removed random objects, whereas REL and UNREL refer to images with removed anchors that are related or unrelated to the action, respectively.

Table S6

Results of Holm-Bonferroni Corrected Post Hoc Estimations for the Model Predicting logRTs in Experiment 1 Including the Accuracy Scores From Experiment 3

Contrast	β	*SE*	*p*
RAND vs REL	0.13	0.04	.004
RAND vs UNREL	-0.01	0.04	.741
REL vs UNREL	-0.14	0.05	.004

Note. *p*-values are Holm-Bonferroni corrected for the three tests. REL = action-related anchor object removed, UNREL = action-unrelated anchor object removed, RAND = action-unrelated non-anchor object removed.

Table S7

Results of Holm-Bonferroni Corrected Post Hoc Estimations for the Model Predicting Accuracies in Experiment 1 Including the Accuracy Scores From Experiment 3

Contrast	β	SE	*p*
RAND vs REL	-1.18	0.26	< .001
RAND vs UNREL	0.27	0.26	.304
REL vs UNREL	1.45	0.32	< .001

Note. *p*-values are Holm-Bonferroni corrected for the three contrasts. Estimates are on the logit scale. REL = action-related anchor object removed, UNREL = action-unrelated anchor object removed, RAND = action-unrelated non-anchor object removed.

Table S8

Results of the Linear Mixed Effects Model Predicting logRT in Experiment 2 Including the Accuracy Scores From Experiment 3

Fixed Effects	β	*SE*	*t*
Intercept	6.65	0.04	150.01
RAND vs REL	0.02	0.01	1.32
RAND vs UNREL	-0.01	0.02	-0.93
RAND vs INCON	0.06	0.01	4.11
Word Length	0.01	0.00	7.79
Accuracy	-0.08	0.02	-4.21

Note. Random intercepts for participants and images are allowed and random slopes for both predictors. $|t| > 2$ is considered significant. RAND refers to images with removed random objects, REL indicates images with action related anchors removed, and UNREL indicates images with action unrelated anchors removed.

Table S9

Results of Holm-Bonferroni Corrected Post Hoc Estimations for the Model Predicting logRTs in Experiment 2 Including the Accuracy Scores From Experiment 3

Contrast	β	*SE*	*p*
RAND vs REL	0.02	0.01	.393
RAND vs UNREL	-0.01	0.02	.393
RAND vs INCON	0.06	0.01	< .001
REL vs UNREL	-0.03	0.02	.393
REL vs INCON	0.04	0.02	.034
UNREL vs INCON	0.07	0.02	< .001

Note. *p*-values are Holm-Bonferroni corrected for the six tests. REL = action-related anchor object removed, UNREL = action-unrelated anchor object removed, RAND = action-unrelated non-anchor object removed, INCON = inconsistent scene category.

Figure S1

Example stimuli depicting a scene in all three object conditions.

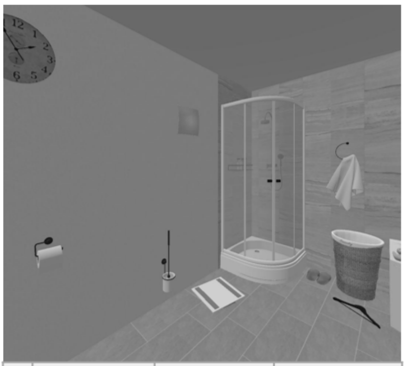

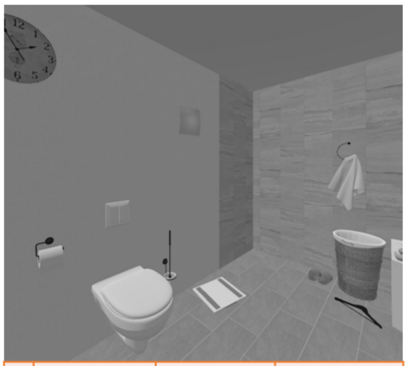

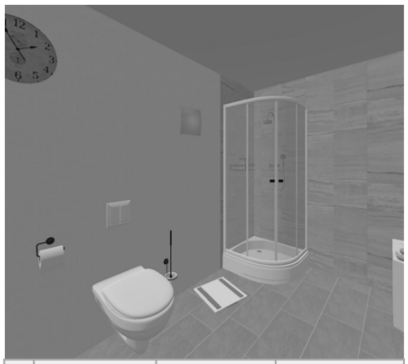

Participant	Block A	Block B	Block C
Participant A	using the toilet	showering	cooking
Participant B	showering	cooking	using the toilet
Participant C	cooking	using the toilet	showering
Participant D	using the toilet	showering	cooking
Participant E	showering	cooking	using the toilet
Participant F	cooking	using the toilet	showering
Participant G	using the toilet	showering	cooking
Participant H	showering	cooking	using the toilet
Participant I	cooking	using the toilet	showering

Note. The stimulus depicts an example scene. For the example action "using the toilet", the stimulus depicts (from left to right) an action related anchor removed (REL), an action unrelated anchor removed (UNREL), and random objects removed (RAND). A participant saw only one version of a stimulus throughout the entire experiment. It was paired with both stimulus specific actions (i.e., "using the toilet" and "showering") and an inconsistent action (e.g., "cooking"). The lower grid shows how the counterbalancing was implemented.
